# Factors Associated with Dental Caries in Primary Dentition in a Non-Fluoridated Rural Community of New South Wales, Australia

**DOI:** 10.3390/ijerph14121444

**Published:** 2017-11-23

**Authors:** Amit Arora, Narendar Manohar, James Rufus John

**Affiliations:** 1 School of Science and Health, Western Sydney University, Locked Bag 1797, Penrith, NSW 2751, Australia; narendar.manohar@hotmail.com (N.M.); rufus.benaud11@gmail.com (J.R.J.); 2 Oral Health Services and Sydney Dental Hospital, Sydney Local Health District, Surry Hills, NSW 2010, Australia; 3 Discipline of Paediatrics and Child Health, Sydney Medical School, Westmead, NSW 2145, Australia; 4 Collaboration for Oral Health Outcomes Research, Translation, and Evaluation (COHORTE) Research Group, Ingham Institute for Applied Medical Research, Locked Bag 7103, Liverpool, NSW 1871, Australia; 5 Capital Markets Cooperative Research Centre, Sydney, NSW 2000, Australia

**Keywords:** dental caries, primary dentition, school children, non-fluoridated, Australia

## Abstract

Dental caries persists as one of the most prevalent chronic diseases among children worldwide. This study aims to determine factors that influence dental caries in primary dentition among primary school children residing in the rural non-fluoridated community of Lithgow, New South Wales, Australia. A total of 495 children aged 5–10 years old from all the six primary schools in Lithgow were approached to participate in a cross-sectional survey prior to implementation of water fluoridation in 2014. Following parental consent, children were clinically examined for caries in their primary teeth, and parents were requested to complete a questionnaire on previous fluoride exposure, diet and relevant socio-demographic characteristics that influence oral health. Multiple logistic regression analysis was employed to examine the independent risk factors of primary dentition caries. Overall, 51 percent of children had dental caries in one or more teeth. In the multiple logistic regression analysis, child’s age (Adjusted Odd’s Ratio (AOR) = 1.30, 95% CI: 1.14–1.49) and mother’s extraction history (AOR = 2.05, 95% CI: 1.40–3.00) were significantly associated with caries experience in the child’s primary teeth. In addition, each serve of chocolate consumption was associated with 52 percent higher odds (AOR = 1.52, 95% CI: 1.19–1.93) of primary dentition caries.

## 1. Introduction

Although dental caries is largely preventable, it is still one of the most prevalent chronic childhood diseases worldwide [1]. It is caused by a complex time-dependent interaction between acid-producing bacteria and fermentable carbohydrates, resulting in demineralisation of inorganic component and dissolution of organic structure of the tooth, compromising the overall structure of tooth and often leading to cavitation [2]. Currently, in high-income countries such as Australia, the prevalence of dental caries has significantly decreased due to increase use of fluorides, improved oral hygiene, and a decreased frequency of sugar consumption [3]. However, its treatment still consumes a high proportion of health resources [4].

Dental caries has a wide spectrum of risk factors ranging from child’s gender, increasing age, lack of fluoride exposure, oral health behaviours, unhealthy dietary lifestyle such as use of sugar-sweetened beverages, low socio-economic status (SES), and maternal oral health [5,6,7]. Dental caries is an important public health issue since its lack of treatment leads to pain, repeated prescription of antibiotics, tooth loss, malnutrition, poor childhood development, low self-esteem, and missed school days [8]. Hence, it compromises a child’s overall quality of life. Also, it has been acknowledged that decay in primary teeth is a strong risk factor of dental caries in the permanent teeth [9].

In the Australian perspective, the 2012–2014 National Child Oral Health Survey (NCOHS) reported that 41.7 percent of children aged 5–10 years suffer from dental caries in their primary teeth. Of note, 27.1 percent of these children had at least one primary tooth with untreated decay, that is, one in every four children in this age group had untreated tooth decay [10]. Furthermore, the national survey highlighted that low parental education, low household income, and residence in rural and/or remote areas were some crucial social determinants of higher tooth decay prevalence in Australian children [10]. Data from New South Wales (NSW) report an average of more than one primary tooth is affected by dental caries [11]. Furthermore, within NSW, the hospitalisation rates for dental procedures (to either remove or restore primary teeth affected by dental caries) illustrate a major variability in terms of remoteness i.e., hospitalisation rates were disproportionately high in remote and very remote areas, followed by inner regional areas, while being lowest in major cities [12]. These estimates are an indicator of higher prevalence of dental caries in infants residing in rural areas compared to urban areas of NSW.

The Centre for Disease Control and Prevention in the United States recognises water fluoridation as one of the major public health achievements of 20th century as it reduces the oral health inequality [13]. It is widely reported that water fluoridation leads to an increase in the proportion of caries-free children by 15.4% [14]. Many research studies have highlighted the protective effects of water fluoridation on reduction of caries prevalence i.e., burden of caries is higher among residents in non-fluoridated communities compared to those living in fluoridated communities [15,16]. Parisotto and colleagues [17] from Brazil reported a higher caries burden among children from non-fluoridated areas in comparison to those in fluoridated areas. A large-scale study done in Queensland, Australia, concluded that children living in long-term fluoridated area had 40 percent lower caries prevalence (RR for dmfs: 0.60 (95% CI: 0.44–0.82) than children in non-fluoridated areas [16]. Mc Lee and Brearley-Messer [18] reported a significant increase in the caries experience of children aged 5–6 years and 11–12 years living in low-fluoridated or non-fluoridated areas as compared to those children who live in fluoridated areas within Australia.

In Australia, as per the NSW *Fluoridation of Public Water Supplies Act 1957* [19], water fluoridation is not mandatory in NSW, and the local government councils hold the decision to fluoridate the water supplies. Efforts by NSW Health, in partnership with area health services, Australian Dental Association and local governments, have provided access to fluoridated water to 96% NSW population, whereas 4% rural communities of NSW still lack water fluoridation, hence rendering them prone to high caries risk [20].

Lithgow Local Government Area (LGA) is a rural township located 145 km west of Sydney, having a resident population of 19,756. Prior to 2014, Lithgow LGA was the only community settlement within the precincts of former Sydney West Area Health Services that did not have access to fluoridated water supply [21]. A study reported a higher caries prevalence among children residing in Lithgow in comparison to those based in adjoining fluoridated communities of Bathurst and Orange [3]. Although some studies have reported on oral health in Lithgow children [3,21,22,23], there is limited evidence on the risk factors of dental caries experience in primary school children in non-fluoridated communities of Australia such as Lithgow. Therefore, the aim of this study was to measure caries experience in primary teeth of school children in Lithgow and to identify the factors related to the caries burden. In addition, this study served as a baseline estimate of caries experience in Lithgow children prior to the introduction of water fluoridation; and will assist in drawing up comparisons with a prospective follow-up study after the introduction of water fluoridation.

The key hypothesis of this study is as follows:

**H_0_**:  Socio-behavioural factors are not associated with dental caries in primary dentition among school children in rural residence.

Which will be tested against the following alternative:

**H_A_**:  Socio-behavioural factors are significantly associated with dental caries in primary dentition among school children in rural residence.

## 2. Materials and Methods

This study is nested within a broader cross-sectional survey involving primary school children of Lithgow, a rural town of NSW, before this region was provided access to water fluoridation in 2014 [21]. The school principals of all six primary schools operating in Lithgow LGA were approached through an invitation letter to be a part of the oral health survey and all agreed to do so. All children aged between 5 and 10 years were invited to participate, and there were no specific selection criteria for inclusion or exclusion, except for the age group and residence. Following the schools’ approval, parents of school children were invited to participate in the oral health survey by providing a take-home information pack comprising of (a) an information statement, (b) a consent form, and (c) a detailed oral health questionnaire. To ensure participation in the study, reminders were sent to parents through school newsletters on weekly basis for a total of four weeks.

For the sample size calculation, we adopted a standard α-error of 5%, a confidence interval level of 95%, and an expected prevalence of about 53% based on the caries prevalence status obtained from New South Wales Child Dental Health Survey for the former Sydney West Area Health Service [24]. The minimum sample size required to provide 80% power (1-β) was estimated to be *n* = 400.

### 2.1. Questionnaire Survey

The survey questionnaire was adapted from the National Child and Oral Health Survey questionnaire to ensure standardised data collection and comparison with 2011 national census reports [24]. The questionnaire consisted of questions related to children’s socio-demographics, oral hygiene and dietary lifestyle, residential movements and dental visiting. Tooth brushing frequency was categorised as brushing with a fluoridated toothpaste once or less than once a day and twice or more daily. The dietary questions explored frequency of consumption of cordial beverages, sweet treats, fruit juices, chocolate and carbonated drinks by the child on a regular day. In addition, parental socio-demographics namely age, country of birth, Indigenous origin, annual household income, education, occupation, dental treatment history, and private health insurance coverage were recorded to investigate if these factors have any impact on child oral health. Parental education was classified into high school education or less and tertiary education; while occupation was categorised into three groups namely managers and professionals, skilled workers; and unemployed and pensioners.

### 2.2. Clinical Examination

Following parental consent, all school children aged between 5 and 10 years underwent a clinical dental examination between August 2006 and November 2006 by two dental practitioners in classrooms of their respective schools. Teeth were examined wet, under a halogen light source, and if necessary, a ball-ended World Health Organization (WHO) probe was used for exploring the teeth surfaces for better assessment. The WHO diagnostic criteria were used for assessing dental caries, which defines a carious tooth as a “cavity into the dentine surface” [25].

Caries prevalence in primary teeth is most commonly represented as the mean number of decayed (d), missing due to decay (m), and filled due to decay (f) teeth, which is termed as “dmft” specifically for the primary dentition [26]. In this study, caries prevalence only for primary dentition is reported for children aged 5–10 years since primary [27] teeth exfoliate with advancing age. Caries prevalence was categorised as no dmft scores and dmft scores ≥1.

### 2.3. Examiner Calibration and Reliability

Caries prevalence scores of primary examiner were calibrated under the supervision of a calibrating examiner on daily basis, while intra- and inter-examiner reliability was assessed through Cohen’s Kappa statistics [28]. The kappa value for intra-examiner reliability was 0.98, and that for inter-examiner reliability was 0.93.

### 2.4. Data Analysis

Statistical Package for Social Sciences (SPSS) version 24 (IBM SPSS Statistics for Windows, Version 24.0. Armonk, NY, USA) was used to manage and analyse data. Descriptive statistics were calculated to generate frequencies, percentages, means, and standard deviations of study variables. The estimated residential population at 30 June 2006 of 5- to 10-year-old children in Lithgow was used to calculate sampling weights for each child [29]. These weights were applied when calculating age-specific indices to produce estimates that were representative of 5- to 10-year-old children in Lithgow [21]. Chi-square tests were used to assess categorical data comparisons, and multiple logistic regression analyses were performed to determine the independent risk factor of dental caries prevalence in Lithgow LGA school children. Only the important variables that were consistently significant across previous studies and reviews [7,9,17] were included in the multiple logistic regression analyses and a backward stepwise model was performed. Multicollinearity tests were carried out using linear regression diagnostics and Pearson’s *r* correlation tests for all the independent covariates. No multicollinearity was present between the predictor variables and therefore all were included in the final regression model, which is the model of best fit. The non-significant variables were sequentially eliminated from the model in a backward stepwise manner. To examine the external validity of the results, the observed population estimate of the Lithgow survey was compared against the 2011 Australian Bureau of Statistics (ABS) census [30] by performing one sample z-tests for proportions.

### 2.5. Ethics Approval

Ethical approval for this dental public health survey was obtained from the Human Research Ethics Committee at the University of Sydney, Western Sydney Area Health Service (HS/pme HREC2006/5/5.16(2354)), Catholic Education Board, and New South Wales Department of Education. A written informed consent to participate was taken from parents/guardians of all children.

## 3. Results

Out of 1000 children, a total of 495 children (49.5% response rate) aged between 5 and 10 years were clinically examined for primary caries after obtaining parental consent. Table 1 shows the descriptive statistics and chi-squared test with *p*-values of the factors that were significantly associated with primary caries. Mean scores were calculated for continuous variables. Among 495 children, it is seen that a significant half of children (50.6 percent) had one or more dmft scores, whereas 49.4 percentage did not have any dmft scores (Table 1). Furthermore, the mean number of serves of chocolate and sugary drinks were found to be higher among children who had one or more dmft scores compared to those who were caries-free (Table 1). In addition, the impact of parents’ oral health and extraction history on their child’s caries status is also clearly evident, as parents’ extraction history had significant association with prevalence of primary caries in their children (Table 1).

Table 2 shows the results of univariate and multivariate analyses. In the univariate analysis, factors such as child’s age, toothbrushing frequency, age when toothbrushing commenced, toothpaste type, past exposure to fluoridated water, chocolate and sugary drinks consumption, parental age and education level, extraction history of both the parents, and household income were significantly associated (*p*-value ≤ 0.05) with caries in the primary dentition in children. Consistent with the univariate analysis, the multivariate logistic regression analysis shows that risk factors such as child’s age, increased chocolate consumption, and extraction history of mothers were significantly associated with primary dentition caries. In terms of child’s age as predictor variable, every year of increased age in child was associated with 30 percent higher odds of primary caries incidence (Odds Ratio (OR) = 1.30, 95% CI: 1.14–1.49) (Table 2). Similarly, each additional serve of chocolate consumption increased the child’s odds of developing caries in the primary dentition by 52 percent (OR = 1.52, 95% CI: 1.19–1.93) (Table 2). Finally, in regards to extraction history of mothers as an independent risk factor, every tooth extracted in the mother was associated with twice increased odds of their child having caries in the primary teeth (OR = 2.05, 6.75% CI: 1.40–3.00).

## 4. Discussion

This study aims to provide a snapshot of socio-demographic and health-behavioural determinants that influence prevalence of dental caries in primary dentition among school children residing in the Lithgow community, NSW. Overall findings from the study show that almost 51 percent of the sample population had at least one or more dmft scores (Table 1). Furthermore, it is observed that almost 67 percentage of children who brushed twice or more daily did not have any dmft scores, whereas 42 percent of children who brushed once or less had one or more dmft scores (Table 1).

In the multiple logistic regression analysis, increase in the child’s age (in years) had significant association with higher odds of primary caries. This could be explained by several plausible mechanisms such as age-related causes, caries progression due to early caries experience, and caries susceptibility. In terms of age-related causes, mixed dentition stage is the period around 6 to 12 years where primary teeth are exfoliated and are replaced by their permanent successors [31]. During this period, there is a high risk of caries incidence due to tender gums, crowding of teeth, and cleaning difficulties compounded by other behavioural factors in children [32]. In addition, studies also report that children who have had early caries experience are at higher risk of developing new cavities as they age [33]. In terms of caries susceptibility, morphology of primary teeth could also be a possible factor as studies report that primary teeth are particularly vulnerable to caries as they have proportionally thinner enamel and dentine compared to permanent teeth, which makes them naturally susceptible to caries development and rapid progression [34,35]. Other factors such as developmental defects and aberrations in the tooth composition are also reported to be significant contributors to development of primary caries [36].

Choice of diet in terms of increased uptake of energy-dense, low-nutritious foods contribute to higher prevalence of dental caries [6,37]. Expectedly in this study, there was a statistically significant association between the serves of chocolate intake and prevalence of caries in the primary dentition. Several studies have reported a direct dose-response relationship and a clear biological plausibility of sugar intake and caries development, where cariogenic bacteria thrive under the presence of fermentable carbohydrates such as sucrose, fructose, and glucose [9,38]. Thus, it is clearly evident that higher chocolate consumption leads to more numbers of decayed teeth consistent with findings from other studies [39,40].

Parents play a crucial role as decision makers in making important health-related choices for their children. Positive attitude and knowledge of parents toward a healthy lifestyle have significant links to their child’s quality of life [41]. In this study, a statistically significant association was observed between mother’s extraction history and their child’s caries status. This is consistent with other studies [42,43] that have also reported that children born to mothers who had high rates of tooth loss had higher odds of having poor oral health. Therefore, it is clearly evident that poor maternal health behaviours and lifestyle choices are directly associated with their child’s health and wellbeing [44].

Although a response rate of 50 percent is considered reasonable for validity of a study, lower response rates do not necessarily result in bias [45]. The Lithgow survey had a response rate of 49.5 percent, which was less than anticipated. Lack of interest in participating in surveys that are not perceived as a salient need in a person’s life or due to the proliferating health literacies previously published by other health research articles might have contributed to the low response rates and might have been due to lack of interest in participating in surveys [46].

In order to clarify the possibility of selection bias and to establish the external validity of the study findings, the observed percentages of selected socio-demographic variables such as parent’s country of birth, Indigenous status, and education levels from this study was compared to the corresponding expected percentages of the 2011 ABS census reports based on selected postcodes of Lithgow region. From Table 3, it is observed that there were no significant differences between the expected population estimates of the census and the observed estimates in the Lithgow survey for factors such as education level and Indigenous status of household. However, it is seen that children of Australian-born parents were 3% over-represented in the survey in comparison to the survey statistics.

This study has both strengths and limitations worth reporting. In terms of strengths, this is one of few studies that has explored the socio-behavioural factors that influence caries in the primary dentition among children residing in rural Australian communities prior to water fluoridation. In terms of limitations, this study used the WHO criteria to examine caries for field or epidemiological surveys. Therefore, only the obvious cavitation seen with the naked eye was recorded on wet tooth surfaces. A drawback of the WHO method is that caries in the enamel cannot be examined, which often requires proper dental armamentarium to isolate or dry the teeth. Furthermore, other limitations include small sample size, sampling bias, and choice of cross-sectional study design renders difficulty in establishing causation and the likely chances of some degree of bias due to the use of self-reported questionnaire [47]. This study failed to mention the presence of other important confounders that could distort the findings. This study analysed the total dmft scores instead of specific decayed teeth scores, which is more indicative of current disease burden and treatment needs. In addition, there is opportunity for future research in this community that could possibly compare the prevalence of caries in the dental caries among the same cohort of children before and after water fluoridation.

## 5. Conclusions

This study provides understanding of the various social determinants and health behavioural factors that are associated with prevalence of caries in primary dentition among children residing in the rural community of Lithgow, Australia. Caries prevalence in the primary dentition of children was associated with age, diet and maternal oral health behaviours, suggesting the need for a more effective oral health promotional programs for this targeted population.

## Figures and Tables

**Table 1:** Socio-behavioural factors influencing caries in primary dentition among primary school children of Lithgow Local Government Area (LGA) (*n* = 495).

Socio-Demographic Factors #	N *	Primary Dentition Caries		Chi Square ^α^	*p* Value
		No dmft Score (n = 245)	One or More dmft Scores (n = 250)		
**Age of the child (years) ^m^**	495	245 (7.58) ^m^	250 (8.14) ^m^		
**Frequency of tooth brushing**	494			4.363	0.037
Once or less		81 (33.1%)	105 (42.2%)		
Twice or more	164 (66.9%)	144 (57.8%)			
**Age when toothbrushing first commenced**	472			4.045	0.044
Less than 12 months		26 (11.1%)	14 (5.9%)		
12 months or more		209 (88.9%)	223 (94.1%)		
**Type of toothbrush used**	492			8.831	0.003
Adult fluoride toothpaste		117 (48.0%)	152 (61.3%)		
Children’s fluoride toothpaste		127 (52.0%)	96 (38.7%)		
**Past exposure to water fluoridation**	495			6.832	0.009
Never		160 (65.3%)	190 (76.0%)		
Previously exposed		85 (34.7%)	60 (24.0%)		
**Serves of sugar-sweetened beverages ©**	488	241 (2.34) ^m^	247 (2.98) ^m^		
**Serves of chocolate per day ^®^**	477	235 (0.81) ^m^	242 (1.08) ^m^		
**Mother’s age (years)**	493			6.023	0.049
40 years and over		52 (21.3%)	69 (27.7%)		
20–29 years		25 (10.2%)	36 (14.5%)		
30–39 years		167 (68.4%)	144 (57.8)		
**Father’s age (years)**	405			7.625	0.022
40 years and over		81 (38.4%)	93 (47.9%)		
20–29 years		8 (3.8%)	14 (7.2%)		
30–39 years		122 (57.8%)	87 (44.8%)		
**Father’s education level**	402			5.977	0.014
University or college degree		47 (22.4%)	25 (13.0%)		
Vocational degree or high school		163 (77.6%)	167 (87.0%)		
**Extractions due to tooth decay in Mother**	495			24.029	<0.001
No extractions		151 (61.6%)	99 (39.6%)		
One or more		94 (38.4%)	151 (60.4%)		
**Extractions due to tooth decay in Father**	402			8.520	0.004
No extractions		117 (55.7%)	79 (44.1%)		
One or more		93 (44.3%)	113 (58.9%)		
**Income of the family**	372			8.642	0.013
More than $100 K		31 (17.6%)	20 (10.2%)		
$40 K–100 K		91 (51.7%)	90 (45.9%)		
Up to $40 K		54 (30.7%)	86 (43.9%)		

Only socio-behavioural factors showing significant associations (*p*-value < 0.05) are shown in the table. * Sample size includes only respondents. ^α^ Pearson’s chi-squared test. ^m^ Mean values are calculated for continuous variables. © (1 serve equals 1 cup–250 mL). ^®^ (1 serve equals 1 chocolate).

**Table 2:** Univariate and multivariate logistic regression analysis of primary dentition caries with non-imputed and imputed models.

Socio-Demographic Factors	Univariate Analysis		Multiple Logistic Regression	
	OR with 95% CI	*p*-Value	AOR with 95% CI	*p*-Value
**Age of the child (years)**	1.30 (1.15, 1.47)	<0.001	1.30 (1.14, 1.49)	<0.001
**Frequency of tooth brushing**			NS	
Once or less ^R^	1.00			
Twice or more	0.67 (0.47, 0.97)	0.037		
**Age when tooth brushing commenced**			NS	
Less than 12 months ^R^	1.00			
12 months or more	1.98 (1.01, 3.89)	0.048		
**Type of toothpaste used**			NS	
Fluoride toothpaste ^R^	1.00			
Children’s toothpaste	0.58 (0.40, 0.83)	0.003		
**Past exposure to water fluoridation**			NS	
Never ^R^	1.00			
Previously exposed	0.59 (0.40, 0.87)	0.009		
**Serves of sugar sweetened beverages per day ©**	1.20 (1.08, 1.33)	<0.001	NS	
**Serves of chocolate per day ^®^**	1.50 (1.19, 1.89)	<0.001	1.52 (1.19, 1.93)	0.001
**Age of Mother **			NS	
40 years and above ^R^	1.00			
20–29 years	1.08 (0.58, 2.02)	0.797 ^NS^		
30–39 years	0.65 (0.42, 0.99)	0.046		
**Age of Father**			NS	
40 years and above ^R^	1.00			
20–29 years	1.52 (0.60, 3.81)	0.368 ^NS^		
30–39 years	0.62 (0.41, 0.93)	0.021		
**Education status of Father**			NS	
University or college degree ^R^	1.00			
Vocational degree or high school	1.92 (1.13, 3.27)	0.016		
**Extractions due to tooth decay in Mother**				
No extractions ^R^	1.00		1.00	
One or more	2.45 (1.70, 3.51)	<0.001	2.05 (1.40, 3.00)	<0.001
**Extractions due to tooth decay in Father**				
No extractions ^R^	1.00		NS	
One or more	1.80 (1.21, 2.67)	0.004		
**Income of the family**			NS	
More than $100 K ^R^	1.00			
$40 K–100 K	1.53 (0.81, 2.88)	0.186		
Up to $40 K	2.46 (1.28, 4.76)	0.007		

Only variables which are statistically significant are shown in the table. CI = Confidence interval. NS = Not-significant. ^R^ Reference category. OR—Odds ratio. AOR—Adjusted odds ratio. © (1 serve equals 1 cup–250 mL). ^®^ (1 serve equals 1 chocolate).

**Table 3:** Population benchmark comparison of demographic characteristics of Lithgow from ABS census 2011 report.

Socio-Demographic Characteristics	Survey Estimate (Observed Percentages) % of Children (95%CI)	Observed *p*-Value	2011 Census Report (Expected Percentages) % of Children
**Country of birth of household ^1^**		<0.001 *	
Overseas	12.4 (9.48–15.31)		16.45
Australia	87.6 (84.69–90.51)		83.55
**Indigenous status of household ^2^**		<0.001 *	
Indigenous	4.1 (2.31–5.79)		5.57
Non-Indigenous	95.9 (94.2–97.68)		94.43
**Highest education level in the household ^3^**		0.268	
University or College degree	27.8 (23.88–31.80)		26.83
High school or vocational training	72.1 (68.19–76.11)		73.17

* Statistically significant at 5% level. ^1^ Children were classified to the overseas born category if they had at least one parent who was born overseas. ^2^ Children were classified to the Indigenous category if they had at least one parent who was Indigenous. ^3^ Children were classified to the University or College degree category if they had at least one parent who had a university or college degree.

## References

[1] Kassebaum N.J., Bernabé E., Dahiya M., Bhandari B., Murray C., Marcenes W. (2015). Global burden of untreated caries: A systematic review and metaregression. J. Dent. Res..

[2] Kumarihamy S.L., Subasinghe L.D., Jayasekara P., Kularatna S.M., Palipana P.D. (2011). The prevalence of early childhood caries in 1–2 years olds in a semi-urban area of Sri Lanka. BMC Res. Notes.

[3] Arora A., Evans R.W. (2011). Dental caries in children: A comparison of one non-fluoridated and two fluoridated communities in NSW. N.S.W. Public Health Bull..

[4] Lagerweij M., van Loveren C. (2015). Declining caries trends: Are we satisfied?. Curr. Oral Health Rep..

[5] Dawani N., Nisar N., Khan N., Syed S., Tanweer N. (2012). Prevalence and factors related to dental caries among pre-school children of saddar town, Karachi, Pakistan: A cross-sectional study. BMC Oral Health.

[6] Hayden C., Bowler J.O., Chambers S., Freeman R., Humphris G., Richards D., Cecil J.E. (2013). Obesity and dental caries in children: A systematic review and meta-analysis. Community Dent. Oral Epidemiol..

[7] Leong P.M., Gussy M.G., Barrow S.-Y.L., de Silva-Sanigorski A., Waters E. (2013). A systematic review of risk factors during first year of life for early childhood caries. Int. J. Paediatr. Dent..

[8] Arora A., Schwarz E., Blinkhorn A.S. (2011). Risk factors for early childhood caries in disadvantaged populations. J. Investig. Clin. Dent..

[9] Çolak H., Dülgergil Ç.T., Dalli M., Hamidi M.M. (2013). Early childhood caries update: A review of causes, diagnoses, and treatments. J. Nat. Sci. Biol. Med..

[10] Ha D.H., Roberts-Thomson K.F., Arrow P., Peres K.G., Do L.G., Do L.G., Spencer A.J. (2016). Children’s oral health status in australia, 2012–2014. Oral Health of Australian Children: The National Child Oral Health Study 2012–2014.

[11] Health Stats NSW (2013). Dental Status of Children by Local Health Districts.

[12] Centre for Epidemiology and Evidence (2014). The Health of Children and Young People in NSW: Report of the Chief Health Officer 2014.

[13] Burt B.A. (2002). Fluoridation and social equity. J. Public Health Dent..

[14] Iheozor-Ejiofor Z., Worthington H., Walsh T., O’Malley L., Clarkson J., Macey R., Alam R., Tugwell P., Welch V., Glenny A. (2015). Water fluoridation for the prevention of dental caries. Cochrane Database Syst. Rev..

[15] Armfield J.M. (2005). Public water fluoridation and dental health in New South Wales. Aust. N. Z. J. Public Health.

[16] Do L., Spencer A. (2015). Contemporary multilevel analysis of the effectiveness of water fluoridation in Australia. Aust. N. Z. J. Public Health.

[17] Parisotto T.M., Fernandes L.M.P., Carvalho F.G.D., Coelho E.D.O., Nobre-dos-Santos M., Oliveira O.M.M., Sponchiado S.R.P. (2010). Dental caries and related factors in Brazilian children from fluoridated and non-fluoridated areas. Rev. Odonto Ciência.

[18] Lee J., Brearley Messer L. (2011). Contemporary fluid intake and dental caries in Australian children. Aust. Dent. J..

[19] Shanti S., Chong G., Blinkhorn A. (2010). Successful fluoride plebiscite in the township of Deniliquin, New South Wales, Australia. J. Public Health Dent..

[20] NSW Ministry of Health Fluoridation Coverage. http://www.health.nsw.gov.au/environment/water/Pages/fluoridation-coverage.aspx.

[21] Arora A., Evans R.W., Sivaneswaran S., Sujeer A., Blinkhorn A. (2010). Parental support for water fluoridation in Lithgow, New South Wales. Aust. Dent. J..

[22] John J.R., Mannan H., Nargundkar S., D’Souza M., Do L.G., Arora A. (2017). Predictors of dental visits among primary school children in the rural Australian community of Lithgow. BMC Health Serv. Res..

[23] Arora A., Evans R.W. (2012). Is the consumption of fruit cariogenic?. J. Investig. Clin. Dent..

[24] Phelan C., Byun R., Skinner J.C., Blinkhorn A.S. (2009). Child dental health survey 2007: A snapshot of the oral health status of primary school-aged children in NSW. N. S. W. Public Health Bull..

[25] World Health Organization (2013). Oral Health Survey: Basic Methods.

[26] Klein H., Palmer C., Knutson J. (1938). Studies on dental caries. I. Dental status and dental needs of elementary school children. Public Health Rep..

[27] Aluckal E., Anzil K., Baby M., George E.K., Lakshmanan S., Chikkanna S. (2016). Association between body mass index and dental caries among anganwadi children of Belgaum city, India. J. Contemp. Dent. Pract..

[28] Cohen J. (1960). A coefficient of agreement for nominal scales. Educ. Psychol. Meas..

[29] Australian Bureau of Statistics (2007). Population by Age and Sex, Australia, 2006.

[30] Australian Bureau of Statistics (2011). Lithgow (C) (LGA) Region Data Summary.

[31] Nanci A. (2013). Physiologic tooth movement: Eruption and shedding. Ten Cate’s Oral Histology.

[32] Lynch R.J.M. (2013). The primary and mixed dentition, post-eruptive enamel maturation and dental caries: A review. Int. Dent. J..

[33] Milsom K.M., Blinkhorn A.S., Tickle M. (2008). The incidence of dental caries in the primary molar teeth of young children receiving National Health Service funded dental care in practices in the North West of England. Br. Dent. J..

[34] Kunin A.A., Evdokimova A.Y., Moiseeva N.S. (2015). Age-related differences of tooth enamel morphochemistry in health and dental caries. EPMA J..

[35] Baginska J., Rodakowska E., Milewski R., Kierklo A. (2014). Dental caries in primary and permanent molars in 7–8-year-old schoolchildren evaluated with Caries Assessment Spectrum and Treatment (CAST) index. BMC Oral Health.

[36] Salanitri S., Seow W.K. (2013). Developmental enamel defects in the primary dentition: Aetiology and clinical management. Aust. Dent. J..

[37] Misra S., Tahmassebi J.F., Brosnan M. (2007). Early childhood caries: A review. Dent. Update.

[38] Sheiham A., James W.P.T. (2014). A reappraisal of the quantitative relationship between sugar intake and dental caries: The need for new criteria for developing goals for sugar intake. BMC Public Health.

[39] Doichinova L., Bakardjiev P., Peneva M. (2015). Assessment of food habits in children aged 6–12 years and the risk of caries. Biotechnol. Biotechnol. Equip..

[40] Mafuvadze B.T., Mahachi L., Mafuvadze B. (2013). Dental caries and oral health practice among 12 year old school children from low socio-economic status background in Zimbabwe. Pan Afr. Med. J..

[41] Vann W.F., Lee J.Y., Baker D., Divaris K. (2010). Oral health literacy among female caregivers: Impact on oral health outcomes in early childhood. J. Dent. Res..

[42] Dye B.A., Hsu K.-L.C., Afful J. (2015). Prevalence and measurement of dental caries in young children. Pediatr. Dent..

[43] Bozorgmehr E., Hajizamani A., Malek Mohammadi T. (2013). Oral health behavior of parents as a predictor of oral health status of their children. ISRN Dent..

[44] Nourijelyani K., Yekaninejad M.S., Eshraghian M.R., Mohammad K., Rahimi Foroushani A., Pakpour A. (2014). The influence of mothers’ lifestyle and health behavior on their children: An exploration for oral health. Iran. Red Crescent Med. J..

[45] Nulty D.D. (2008). The adequacy of response rates to online and paper surveys: What can be done?. Assess. Eval. High. Educ..

[46] Galea S., Tracy M. (2007). Participation rates in epidemiologic studies. Ann. Epidemiol..

[47] Delgado-Rodríguez M., Llorca J. (2004). Bias. J. Epidemiol. Community Health.

